# Draft Genome Sequence of *Mycolicibacterium* sp. Strain NCC-Tsukiji, Isolated from Blood Culture of a Patient with Malignant Lymphoma

**DOI:** 10.1128/MRA.01575-18

**Published:** 2019-04-04

**Authors:** Hanako Fukano, Mitsunori Yoshida, Michi Shouji, Shunsuke Hatta, Dai Maruyama, Koji Izutsu, Mika Shiotsuka, Yoshitoshi Ogura, Tetsuya Hayashi, Naoki Hasegawa, Satoshi Iwata, Yoshihiko Hoshino

**Affiliations:** a Department of Mycobacteriology, Leprosy Research Center, National Institute of Infectious Diseases, Tokyo, Japan; b Department of Clinical Laboratory, National Cancer Center Hospital, Tokyo, Japan; c Department of Hematology, National Cancer Center Hospital, Tokyo, Japan; d Department of Infectious Diseases, National Cancer Center Hospital, Tokyo, Japan; e Department of Bacteriology, Faculty of Medical Sciences, Kyushu University, Fukuoka, Japan; f Center for Infectious Diseases and Infection Control, Keio University School of Medicine, Tokyo, Japan; University of California, Riverside

## Abstract

A nonidentifiable mycolicibacterium was isolated from a malignant lymphoma patient treated with intensive chemoimmunotherapy. Multilocus sequence analysis showed that this isolate was close to “Mycolicibacterium (Mycobacterium) ratisbonense,” but the details of this species were still unknown. Here, we report the draft genome sequence data of *Mycolicibacterium* sp. strain NCC-Tsukiji.

## Abstract (precis)

A nonidentifiable mycolicibacterium was isolated from a malignant lymphoma patient treated with intensive chemoimmunotherapy. Multilocus sequence analysis showed that this isolate was close to “Mycolicibacterium (Mycobacterium) ratisbonense,” but the details of this species were still unknown.

## ANNOUNCEMENT

The Mycolicibacterium mucogenicum group (M. mucogenicum, M. aubagnense, and M. phocaicum) comprises rapidly growing acid-fast bacteria (1, 2). This species is commonly isolated from water-associated equipment, such as the drinking water supply (3, 4). The risk factor for infection with M. mucogenicum is immune suppression, including anticancer chemotherapy (5–7). Significant clinical cases are catheter-related bloodstream infections (6, 8). Long-term placement of a catheter and bathing with contaminated water can be a risk for infection (5–7, 9).

We isolated M. mucogenicum-related species from a malignant lymphoma patient treated with a long-time course of intensive chemoimmunotherapy. The agent was detected 6 days after culturing blood using the BD Bactec FX system with BD Bactec Plus aerobic medium (Becton, Dickinson, USA). A single clone was isolated on sheep blood agar (Nissui, Japan). First, the isolated colony was submitted to matrix-assisted laser desorption–ionization time of flight mass spectrometry (MALDI-TOF MS) (Bruker Daltonics, Germany) for identification. In addition, partial 16S rRNA and *rpoB* genes were amplified by PCR and sequenced with an ABI Prism 3730 DNA analyzer (Applied Systems, USA). NCBI BLAST analysis was then performed to determine the identity of each strain with the BLASTN algorithm using the default parameters. The results of MALDI-TOF MS indicated that the agent was *Mycolicibacterium mucogenicum*, but the results of the BLAST search using a partial sequence of multilocus genes (a 16S rRNA gene and an *rpoB* gene) showed it shared high similarity with “*Mycolicibacterium* (*Mycobacterium*) *ratisbonense*,” which was isolated from a human urine sample. The 16S rRNA gene and *rpoB* gene sequences of NCC-Tsukiji shared 100% (1,442/1,442 bp) and 99% (632/633 bp) identities with the sequences of *M. ratisbonense* (the GenBank/EMBL/DDBJ accession numbers for the two partial genes are AF055331 and LT718451) (10).

The strain was inoculated on Middlebrook 7H9 broth supplemented with 10% oleic acid-albumin-dextrose-catalase enrichment and incubated at 30°C for 7 days (11–14). Genomic DNA extractions were performed using a NucleoSpin plant II kit (Macherey-Nagel). A DNA sequencing library was prepared using a Nextera XT DNA sample prep kit (Illumina, Inc., San Diego, CA) according to the manufacturer’s instructions. The genomic sequencing was obtained by using Illumina 2 × 150-bp paired-end reads. Low-quality reads (quality score, 15), the quality score of short reads (length, <25), and adaptors were removed, and the paired-end reads were assembled using the Platanus version 1.1 package with default parameters. Genome quality was assessed using the QUAST Web interface (15). Automated annotation was carried out with the DDBJ Fast Annotation and Submission Tool (DFAST) (https://dfast.nig.ac.jp/). Average nucleotide identity (ANI) was calculated by JSpeciesWS version 1.2.1 (16, 17).

The total number of paired-end reads was 429,643. The assembly comprised 159 contigs with a total length of 5,752,239 bp and an *N*_50_ value of 78,866 bp. The GC content was 67.12%.

A total of 5,555 protein-coding sequences (CDS), 2 rRNAs, and 68 tRNAs were predicted. The ANI value compared to M. mucogenicum strain CSUR P2099 (GenBank accession number NZ_CYSI00000000) was 91.26%.

### Data availability.

The scaffold sequences and annotations of *Mycolicibacterium* sp. strain NCC-Tsukiji were deposited at DDBJ/EMBL/GenBank under the accession number BHVW00000000. The version described in this paper is the first version, BHVW01000000. Raw sequence data for strain NCC-Tsukiji were deposited under SRA accession number PRJDB7019.
